# Supplementary material for: The Systems Biology Research Tool: evolvable open-source software
Source: BMC Syst Biol. 2008 Jun 29;2:55. doi: 10.1186/1752-0509-2-55 (PMC2446383; doi:10.1186/1752-0509-2-55)
Supplement: Additional file 1 — SBRT Archive. An archive of the current version of the Systems Biology Research Tool. [file 1752-0509-2-55-S1.zip › sbrt-1.4.0/doc/developers_guide/api/sbrt/shell/io/class-use/ComoFile.html]

Uses of Class sbrt.shell.io.ComoFile


|  |  |  |
| --- | --- | --- |
| **Overview** \| **Package** \| **Class** \| **Use** \| **Tree** \| **Deprecated** \| **Index** \| **Help** | |  |
| PREV   NEXT | **FRAMES**    **NO FRAMES**     **All Classes** |  |


---


## **Uses of Class sbrt.shell.io.ComoFile**

| Packages that use ComoFile | |
| --- | --- |
| **sbrt.shell.io** | Provides classes and interfaces for reading and writing files. |
| **sbrt.shell.mng.fba** | Provides classes and interfaces for creating Flux Balance Analysis process managers. |
| **sbrt.shell.mng.geom** | Provides classes and for creating geometry process managers. |

Uses of ComoFile in sbrt.shell.io

| Methods in sbrt.shell.io that return ComoFile | |
| --- | --- |
| `ComoFile<K,V>` | `ComoFileSource.next()`             Returns the next column-oriented map output file with the next name returned by the file name source. |

Uses of ComoFile in sbrt.shell.mng.fba

| Methods in sbrt.shell.mng.fba that return ComoFile | |
| --- | --- |
| `static ComoFile<java.lang.String,Interval>` | `SimpleFluxIntervalManager.getMultiResultsOutputFile(ManagerInput<java.lang.String,java.lang.String> input, java.io.PrintWriter out, Fluxome fluxome)`             Returns an empty output file that is suitable for storing multiple sets of flux interval results. |
| `static ComoFile<java.lang.String,Interval>` | `SimpleFluxIntervalManager.getMultiResultsOutputFile(java.lang.String fileName, java.util.Set<java.lang.String> headers, Fluxome fluxome)`             Returns an empty output file that is suitable for storing multiple sets of flux interval results. |
| `static ComoFile<java.lang.String,java.lang.Double>` | `ExtremeCurrentManager.getOutputFile(ManagerInput<java.lang.String,java.lang.String> input, java.io.PrintWriter out, Fluxome fluxome, java.util.Set<java.lang.String> rxnNames)`             Returns an output file to which extreme currents can be written. |
| `static ComoFile<java.lang.String,java.lang.Double>` | `ExtremeCurrentManager.getOutputFile(java.lang.String fileName, java.io.PrintWriter out, Fluxome fluxome, java.util.Set<java.lang.String> rxnNames)`             Returns an output file to which extreme currents can be written. |

| Methods in sbrt.shell.mng.fba that return types with arguments of type ComoFile | |
| --- | --- |
| `static MultiOutputFiles<ComoFile<java.lang.String,java.lang.Double>,java.util.Map<? extends java.lang.String,? extends java.lang.Double>>` | `SvCdHarFluxVectorManager.getMulitComoFiles(ManagerInput<java.lang.String,java.lang.String> input, java.io.PrintWriter out, java.util.Iterator<java.lang.String> fileNameSource, Fluxome fluxome)`             Returns a source of output files. |

Constructors in sbrt.shell.mng.fba with parameters of type ComoFile

`ExtremeCurrentManager.Rm(ComoFile<java.lang.String,java.lang.Double> output)`

Uses of ComoFile in sbrt.shell.mng.geom

| Methods in sbrt.shell.mng.geom that return ComoFile | |
| --- | --- |
| `static ComoFile<java.lang.String,java.lang.Double>` | `CdHarManager.getOutputFile(ManagerInput<java.lang.String,java.lang.String> input, java.io.PrintWriter out, java.util.Set<java.lang.String> headers)`             Returns an output file with the name provided in the manager input. |
| `static ComoFile<java.lang.String,java.lang.Double>` | `CdHarManager.getOutputFile(java.lang.String fileName, java.io.PrintWriter out, java.util.Set<java.lang.String> headers)`             Returns an output file with the provided name. |
| `static ComoFile<java.lang.String,java.lang.Double>` | `CdHarManager.getOutputFile(java.lang.String fileName, java.util.Set<java.lang.String> headers)`             Returns an output file with the provided name. |

---


|  |  |  |
| --- | --- | --- |
| **Overview** \| **Package** \| **Class** \| **Use** \| **Tree** \| **Deprecated** \| **Index** \| **Help** | |  |
| PREV   NEXT | **FRAMES**    **NO FRAMES**     **All Classes** |  |


---
